# Predictors of HIV Vertical Transmission in Exposed Infants in Northwest, Ethiopia: A Retrospective Cohort Study

**DOI:** 10.1002/hsr2.71766

**Published:** 2026-01-20

**Authors:** Amare Genetu Ejigu, Tilahun Degu, Ahmed Fentaw Ahmed, Abathun Temesgen, Almaw Genet Yeshiwas, Abebaw Molla Kebede, Gashaw Melkie Bayeh, Chalachew Yenew, Asaye Alamneh Gebeyehu, Biresaw Wassihun Alemu, Zeamanuel Anteneh Yigzaw, Habitamu Mekonen, Tewodros Worku Bogale, Rahel Mulatie Anteneh, Anley Shiferaw Enawgaw, Getasew Yirdaw, Roza Belayneh Desalegn, Berhanu Abebaw Mekonnen, Meron Asmamaw Alemayehu, Sintayehu Simie Tsega

**Affiliations:** ^1^ Department of Midwifery, College of Medicine and Health Sciences Injibara University Injibara Ethiopia; ^2^ Department of Public Health, College of Medicine and Health Sciences Injibara University Injibara Ethiopia; ^3^ Department of environmental health, college of medicine and health science Injibara University Injibara Ethiopia; ^4^ Department of Environmental Health Sciences, Public Health, College of Health Sciences Debre Tabor University Debre Tabor Ethiopia; ^5^ Depatment of Public Health, College of health science Debre Tabor University Debre Tabor Ethiopia; ^6^ Department of Health Promotion and Behavioral Sciences, School of Public Health, College of Medicine and Health Sciences Bahir Dar University Bahir Dar Ethiopia; ^7^ Department of Human Nutrition, Collage of Health Science Debre Markos University Debre Markos Ethiopia; ^8^ Department of Public Health, College of Health Sciences Debre Markos University Debre Markos Ethiopia; ^9^ Department of Environmental Health Science, College of Medicine and Health Sciences Debre Markos University Ethiopia; ^10^ Department of Nutrition and Dietetics, School of Public Health, College of Medicine and Health Sciences Bahir Dar University Bahir Dar Ethiopia; ^11^ Department of Epidemiology and Biostatistics, Institute of Public Health, College of Medicine and Health Sciences University of Gondar Gondar Ethiopia; ^12^ Department of Medical Nursing, School of Nursing, College of Medicine and Health Science University of Gondar Gondar Ethiopia

**Keywords:** HIV exposed infant and Ethiopia, mother‐to‐child transmission of HIV, vertical transmission of HIV

## Abstract

**Background and Aims:**

Although the global plan aimed to eliminate mother‐to‐child transmission of HIV by 2015, an estimated 130,000 children were newly infected, and 84,000 children died from HIV related causes in 2022. In Ethiopia, 3200 children became newly infected and 1,900 children died from HIV related causes in 2022. A new target has now been set for 2030. Vertical transmission remains the predominant route of pediatric HIV infection. This study aimed to assess HIV vertical transmission and its predictors among HIV‐exposed infants in Northwest Ethiopia.

**Methods:**

A retrospective cohort study was conducted among 480 mother–infant pairs enrolled in the PMTCT program. Data were analyzed using R version 4.3.2. To account for missing values, multiple imputations were performed prior to bivariate and multivariable logistic regression analyses. Associations with a *p*‐value < 0.05 were considered statistically significant.

**Results:**

The rate of vertical transmission of HIV was 3.6%. Mothers residing outside the catchment area (AOR = 4.74; 95% CI: 1.17–20.63) and those not receiving antiretroviral therapy (AOR = 6.82; 95% CI: 1.33–38.73) had significantly higher odds of vertical HIV transmission. Conversely, maternal HIV diagnosis prior to pregnancy (AOR = 0.08; 95% CI: 0.009–0.84), receiving Nevirapine prophylaxis (AOR = 0.14; 95% CI: 0.03–0.65), and delivery at a health facility (AOR = 0.08; 95% CI: 0.01–0.66) were significantly protective against mother‐to‐child transmission.

**Conclusion:**

The rate of vertical HIV transmission was found to be high. Therefore, we recommend further PMTCT service expansion; focus on maternal HIV diagnosis in the preconception period; improve provision of infant Nevirapine prophylaxis and maternal ART help to minimize HIV vertical transmission. Reduce home delivery also helps to decrease HIV vertical transmission.

## 1 Introduction

Mother‐to‐child transmission (MTCT), also referred to as vertical transmission, is the transmission of human immunodeficiency virus (HIV) from an infected mother to her child during pregnancy, labor and delivery, or through breastfeeding in the postpartum period [1]. It is estimated that 73%–95% of pediatric HIV infections result from vertical transmission [2, 3].

Despite the global plan to eliminate new HIV infections among children by 2015 [4, 5], an estimated 1.5 million children were living with HIV worldwide, and 130,000 became newly infected in 2022 [6, 7]. In Ethiopia, approximately 42,000 children were living with HIV, and 3200 became newly infected in 2022 [8, 9]. In 2018, an estimated 14.8 million children were HIV‐exposed globally, of whom 13.2 million resided in sub‐Saharan Africa (SSA) [10]. In response, a new target has been set for 2030, underscoring the urgent need to strengthen interventions in high‐burden regions such as SSA [11].

The HIV/AIDS epidemic has had a profound impact on child health, well‐being, and survival [12, 13]. In 2022, approximately 84,000 children worldwide died from HIV‐related causes [14], while in Ethiopia, approximately 1900 child deaths were attributed to HIV in 2021 [8, 9]. Without antiretroviral therapy (ART), 15%–30% of vertically infected infants die within their first year of life [1]. Previous studies have shown that HIV infection increases the risk of perinatal and childhood mortality [15, 16]. The incidence of opportunistic infections has also risen among people living with HIV, with common conditions in children including tuberculosis, herpes zoster, chronic diarrhea, bacterial and pneumocystis pneumonia, oral candidiasis, Kaposi's sarcoma, and cryptococcal meningitis [17, 18]. Furthermore, the lifetime HIV‐related medical cost for a person living with HIV has been estimated at approximately USD 420,285 (about 55.7 million ETB, based on the 2025 average exchange rate of 1 USD ≈ 132.6 ETB), assuming an average of 29.3 years on ART [19].

The rate of MTCT of HIV can reach approximately 45% in the absence of any intervention [20, 21]. However, with the implementation of effective prevention strategies, this rate can be reduced to below 1% [3, 22]. In 2022, an estimated 1.2 million pregnant women worldwide were living with HIV, of whom about 82% received ART for the prevention of mother‐to‐child transmission (PMTCT) [23]. In SSA, ART coverage among pregnant women was 69.5% in 2019 [24], while in Ethiopia it was reported at 78% in 2021 [8].

A meta‐analysis conducted in East Africa reported an overall MTCT prevalence of 7.68% [25]. Studies from other settings showed varying rates: 2%–7.3% in Brazil [26, 27], 8.9% in Vietnam [28], and 1.54%–9.3% across different African countries [15, 29, 30, 31, 32, 33, 34]. In Ethiopia, meta‐analyses have estimated the prevalence of MTCT to range between 9.93% and 11.4% [35, 36]. Findings from individual studies conducted in different parts of Ethiopia have reported prevalence rates ranging from 3.8% to 15.7% [37, 38, 39, 40].

Previous studies have identified several factors associated with MTCT of HIV, including maternal residence, place of delivery, antenatal care (ANC) follow‐up, maternal ART use, infant ARV prophylaxis, mixed feeding, and maternal WHO clinical stage [25, 32, 36, 38, 40, 41]. However, the prevalence and determinants of MTCT have not been consistent across health facilities or socio‐demographic groups. The Awi Zone, located in Northwest Ethiopia, is a predominantly rural area with limited access to healthcare services, including PMTCT programs. Although national‐level data on HIV transmission are available, no prior study has specifically examined MTCT in this zone. Given the health system challenges in the region and the need for localized evidence, this study was designed to establish baseline estimates and identify factors associated with MTCT in the Awi Zone. Therefore, the objective of this study was to assess the rate and determinants of MTCT of HIV among HIV‐exposed infants (HEI) in public health facilities of Awi Zone, Northwest Ethiopia.

## 2 Methods

### 2.1 Study Setting and Design

An institution‐based retrospective cohort study was conducted among HIV‐positive mother–infant pairs enrolled in PMTCT services at 15 public health facilities in the Awi Zone, Northwest Ethiopia. The zone had an estimated population of 1,288,790 in 2021, of whom 643,276 were female [42]. Administratively, it comprises 9 districts (rural woredas) and 6 town administrations, and is served by 46 health centers and 5 hospitals, most of which provide PMTCT services. The study included mother–infant pairs enrolled between July 2015 and July 2020. Infants who were transferred out, lost to follow‐up, or who died before outcome ascertainment were excluded.

### 2.2 Sample Size Determination

The required sample size was determined using a single population proportion formula, with the following assumptions: a 95% confidence level, a 3% margin of error, and an estimated vertical HIV transmission rate of 11.4% (*p*), obtained from a previous study [36]. To account for potential non‐response, 10% was added, giving the final adjusted sample size [43].

$$n=\frac{\left(Z^{2}*\;p*\;\left(1-p\right)\right)}{\left(d^{2}\right)}$$

Where:

- *n* = required sample size
- Z = standard normal value (1.96 for 95% confidence)
- p = estimated prevalence of vertical HIV transmission (0.114)
- d = margin of error (0.03)

$$n=(1.96^{2}*\;0.114*\;(1-0.114))/(0.03^{2})$$

n=431.13≈432

**Non‐response Adjustment:**

$$n_{\text{final}}=n/\left(1-r\right)$$

Where r = non‐response rate (0.10 for 10%)

$$n_{\text{final}}=432/\left(1-0.10\right)=432/0.90=480$$

**Final Sample Size:** 480 mother–infant pairs.

### 2.3 Sampling Technique

From the nine districts and six town administrations in the Awi Zone, four districts (Banja, Ankesha Guagusa, Dangila, and Zigem) and three town administrations (Chagni, Injibara, and Dangila) were randomly selected using the lottery method, ensuring representation of at least 50% of the districts and town administrations in the zone. Within these selected areas, there were 26 public health facilities, of which 15 provided PMTCT services. All 15 health facilities offering PMTCT services were included in the study. The study population comprised all HIV‐positive mother–infant pairs enrolled in PMTCT services at these facilities between July 2015 and July 2020.

### 2.4 Operational Definition

**HIV positive child:** An infant whose dried blood spot (DBS) test result indicated HIV infection during the 24‐month follow‐up period, as documented on the infant registration card. HIV infection was confirmed either at 6 weeks or later using deoxyribonucleic acid–polymerase chain reaction (DNA‐PCR) virology tests, or at 18 months or older using a DNA‐PCR test or rapid antibody test performed at least 6 weeks after breastfeeding cessation [44].

**HIV‐Exposed infant:** An infant born to an HIV‐positive mother or one who tested HIV antibody positive before 18 months of age [44].

**Health facility catchment area:** The geographical area surrounding a health facility that defines the population utilizing some or all of its services [45].

**Mixed feeding:** HEI received both breast milk and other food or fluid within 6 months of birth.

### 2.5 Data Collection Procedure and Quality Assurance

Data were collected from both infant and maternal records, including cards, forms, and registers. The data extraction tool was adapted from the standardized PMTCT documentation system, which included:

- **Cards:** Integrated ANC, labor and delivery, newborn, postnatal, HEI follow‐up, and women's cards.
- **Forms:** ART intake form, HIV chronic care follow‐up card, and transfer‐out form.
- **Registers:** ANC register, labor and delivery register, and mother–baby pair cohort register [44].

To ensure data quality, several measures were undertaken. Technical training was provided to data collectors and supervisors prior to data collection. The data extraction format was pre‐tested in health facilities outside the study sites, but with similar characteristics to the target population, in order to refine the tool. Data collection was carried out by trained midwives and supervised by senior health staff. Supervisors checked the completeness, accuracy, and consistency of the collected data on a daily basis.

### 2.6 Data Analysis

After data collection, questionnaires were coded and entered into Epi‐Data version 3.5 and then exported to R version 4.3.2 for analysis. To address missing data, multiple imputations by chained equations (MICE) was applied under the assumption of missing at random (MAR) [46]. Apart from the 31 HEI who were lost to follow‐up, died, or were transferred out after enrollment in PMTCT services, no outcome data were missing; therefore, outcomes were not imputed.

Bivariate and multivariable logistic regression analyses were performed to identify factors associated with MTCT of HIV. Adjusted odds ratios (AOR) with 95% confidence intervals (CI) were calculated, and statistical significance was declared at a *p*‐value < 0.05.

## 3 Result

### 3.1 Socio‐Demographic Characteristics of Respondents

A total of 449 HEI were included in the analysis, and this resulting in a response rate of 93.5% of the eligible participants. Among them, 91 (20.3%) resided in rural areas, and 352 (78.4%) lived within the catchment area of PMTCT services. With regard to maternal characteristics, the majority, 302 (67.3%), were in the age group of 25–34 years, and 367 (81.7%) were Orthodox Christian. In terms of marital status, 107 (23.8%) were divorced or widowed. Concerning educational status, 122 (27.2%) had no formal education, while 217 (48.4%) were housewives (Table 1).

**Table 1:** Socio‐demographic characteristics of HEI and their mothers, in selected public health facilities of Awi Zone, Ethiopia (*n* = 449), July 2015–2020.

Variables	Variable categories	Frequency	Percentage
Age of women	15–24	93	20.7
	25–34	302	67.3
	≥ 35	54	12
Religion	Orthodox	367	81.7
	Protestant	21	4.7
	Muslim	59	13.1
	Catholic	2	0.5
Women residence	Rural	91	20.3
	Urban	358	79.7
Women reside within catchment area	Yes	352	78.4
	No	97	21.6
Women marital status	Single	61	13.6
	Married	281	62.6
	Divorced/Widowed	107	23.8
Women educational status	No formal education	122	27.2
	Primary (Grade1–8)	112	24.9
	Secondary (9–12)	142	31.6
	Secondary & above	73	16.3
Women occupational status	House wife	217	48.4
	Government employee	98	21.8
	Merchant	134	29.8

### 3.2 HIV Care and Support

All mother of HEI were enrolled in HIV care and support. Among women enrolled in PMTCT services, 403 (89.7%) received ART during pregnancy and 359 (80.0%) during labor and delivery. The majority, 398 (88.6%), were classified as WHO clinical stage I. A total of 84 (18.7%) women had a CD4 count below 200 cells/mm³.

Regarding the infants, 411 (91.5%) were diagnosed at 6 weeks of age, and 393 (87.5%) received Nevirapine prophylaxis. Maternal syphilis co‐infection was identified in 15 (3.3%) women. Among fathers, 383 (94.6%) were HIV‐positive. In terms of delivery, 437 (97.3%) mothers gave birth at health facilities. Almost all infants, 445 (99.1%), were exclusively breastfed during the first 6 months of life, and 137 (30.5%) were given replacement feeding after 6 months (Table 2).

**Table 2:** HIV care and support of HEI and their mothers, in selected public health facilities of Awi Zone, Ethiopia (*n* = 449), July 2015–2020.

Variables	Variable categories	Frequency	Percentage
On ART	Yes	403	89.7
	No	46	10.3
ART during labor and delivery	Yes	359	80
	No	90	20
Maternal clinical stage	I	398	88.6
	II	34	7.6
	III	14	3.1
	IV	3	0.7
Maternal CD4 count	< 200	84	18.7
	200–350	65	14.5
	> 350	300	66.8
Infant receive nevirapine	Yes	393	87.5
	No	56	12.5
Adherence to ART	Good	380	94.3
	Fair	19	4.7
	Poor	4	1
Father's HIV status	Positive	383	94.6
	Negative	22	5.4
Maternal syphilis	Positive	15	3.3
	Negative	434	96.7
Infant feeding for 1st 6 months	EBF	445	99.1
	ERF	2	0.45
	Missed feeding	2	0.45
Infant feeding after 6 months	BF with complement	312	69.5
	Replacement feeding	137	30.5
Place of delivery	Same health facility	283	63
	Other health facility	154	34.3
	Home	12	2.7
Infant age at diagnosis	At 6 weeks	411	91.5
	After 6 weeks	38	8.5
Age at discharge from program (in month)	< 12	28	6.2
	12–18	99	22
	18	49	11
	> 18	273	60.8

Abbreviations: EBF = exclusive breast feeding, ERF = exclusive replacement feeding.

### 3.3 Timing of Maternal HIV Diagnosis

**Figure 1:**
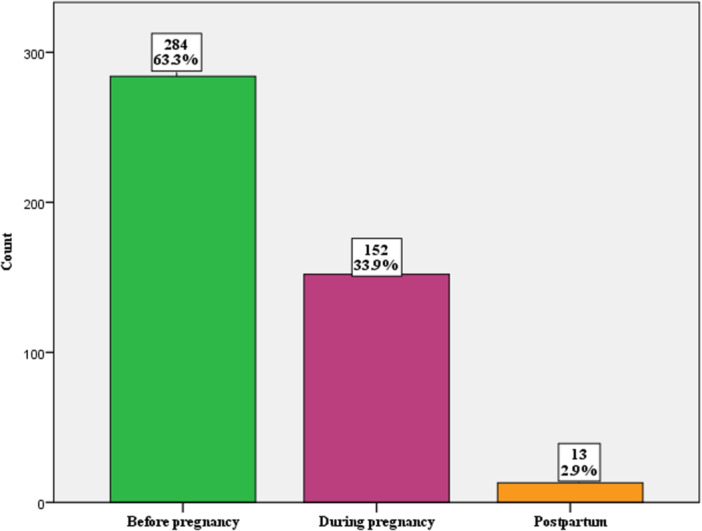
Distribution of the timing of maternal HIV diagnosis among study participants (*N* = 449).

A total 284 (63.3%) of women knew their HIV status before pregnancy (Figure 1).

### 3.4 Rate of MTCT of HIV

**Figure 2:**
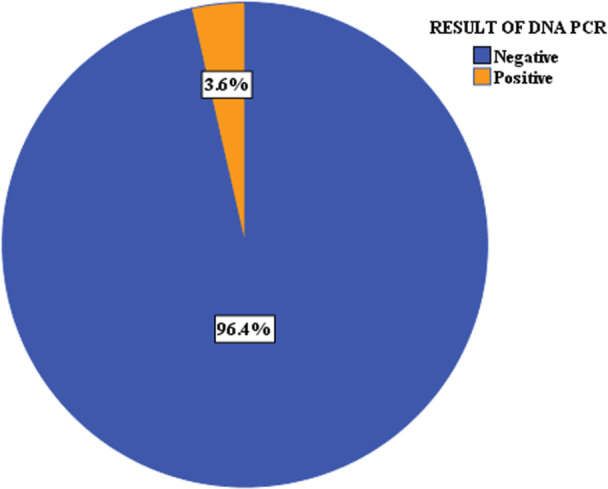
Proportion of HEI by DNA‐PCR test result (*N* = 449).

The rate of HIV transmission among HEI, as confirmed by DNA‐PCR testing, was 16 (3.6%) (Figure 2).

### 3.5 Factors Associated With MTCT of HIV

Residence outside the catchment area [AOR = 4.74 (1.17, 20.63)], maternal HIV diagnosis before pregnancy [AOR = 0.08 (0.009, 0.84)], children born to mothers who did not receive ART [AOR = 6.82 (1.33, 38.73)], facility‐based delivery [AOR = 0.08 (0.01, 0.66)] and infant receipt of Nevirapine prophylaxis [AOR = 0.14 (0.03, 0.65)] were significantly associated with vertical transmission of HIV (Table 3).

**Table 3:** Factors associated with MTCT of HIV among infants born to HIV positive mothers in selected public health facilities of Awi Zone, Ethiopia (*n* = 449), July 2015–2020.

		DBS result				
Variables		Yes (*n* = 16)	No (*n* = 433)	COR (95.0% CI)	AOR (95.0% CI)	*p* value
Residence	Rural	7	84	3.23 (1.12, 8.92)	1.16 (0.26, 4.52)	0.83
	Urban	9	349	1		Ref
Reside within catchment area	Yes	6	346	1		Ref
	No	10	87	6.62 (2.39, 19.94)	4.74 (1.17, 20.63)	0.03*
Timing of diagnosis	ANC and delivery	7	145	0.11 (0.03, 0.47)	0.09 (0.008, 0.83)	0.04*
	Postpartum	4	9	1		Ref
	Prior to pregnancy	5	279	0.04 (0.009, 0.185)	0.08 (0.009, 0.84)	0.03*
On ART	Yes	10	393	1		Ref
	No	6	40	5.89 (1.92, 16.74)	6.82 (1.33, 38.73)	0.02*
Infant age at diagnosis	At 6 weeks	10	401	7.52 (2.43, 21.62)	2.80 (0.52, 13.72)	0.21
	After 6 weeks	6	32	1		Ref
Feeding after 6 months	BF with complementary	9	128	3.06 (1.12, 8.74)	2.89 (0.70, 14.33)	0.15
	Replacement Feeding	7	305	1		Ref
Place of delivery	Health facility	9	274	0.10 (0.02, 0.5)	0.08 (0.01, 0.66)	0.02*
	Other health facility	4	150	0.08 (0.015, 0.46)	0.11 (0.01, 1.00)	0.05*
	Home	3	9	1		Ref
Age at discharge from program (in month)	< 12	3	25	1		Ref
	12–18	5	94	0.44 (0.10, 2.28)	2.80 (0.27, 53.63)	0.43
	18	2	47	0.35 (0.04, 2.27)	0.51 (0.03, 9.86)	0.70
	> 18	6	267	0.19 (0.05, 0.93)	1.26 (0.16, 19.72)	0.90
Nevirapine	Yes	9	384	0.16 (0.06, 0.48)	0.14 (0.03, 0.65)	0.01*
	No	7	49	1		Ref

Key * = *p* < 0.05: Statistically significantly variables, Ref = Reference

## 4 Discussion

Timely preventive treatment and early diagnosis of HEI is required to reduce MTCT of HIV. DNA/PCR testing has been expanded in Ethiopia as a standard testing mechanism for early HIV diagnosis. Therefore, monitoring and evaluating the rate of MTCT of HIV among HEI is a key indicator for understanding the performance of the PMTCT program. In the Awi Zone, DNA/PCR testing for HEI has been implemented since 2014, with progressively increasing coverage through government‐supported initiatives. At the time of this study, most health facilities providing PMTCT services had access to DNA/PCR testing; however, coverage varied between rural and urban settings. Therefore, this study aimed to assess the rate of HIV vertical transmission and its associated factors among HEI in the Awi Zone, Northwest Ethiopia.

According to our study, the rate of HIV vertical transmission was 3.6% [CI= (1.84, 5.27)]. This finding is consistent with studies conducted in Dessie Town (3.8%) and Brazil (2%) [26, 40]. However, it is higher than recent reports from Nigeria (1.54%), Zimbabwe (1.55%) and Rwanda (1.58%) [29, 30, 31]. Conversely, the rate observed in this study is lower than findings from East and West Gojjam Zones (5.9%), Dire Dawa City (15.7%), and Gondar University referral hospital (10%) [37, 38, 39], Uganda (6.5%) [32], Kenya (8.9%) [34], Tanzania (9.3%) [15], Brazil (7.3%) [27], Cameron (7.1%) [33], and Vietnam(8.9%) [28]. Moreover, it is lower than pooled estimates from meta‐analyses conducted in Ethiopia (9.93% and 11.4%) [35, 36] and East Africa (7.68%) [25]. These differences may be attributed to variations in the geographic distribution of HIV prevalence among women, timing of the studies, and the availability and quality of HIV care and support services for both mothers and HEI.

In our study, majority of women (63.2%) knew their HIV status before pregnancy, and the rate of MTCT was 3.6%. In East and West Gojjam, only 10.5% of women knew their HIV status before pregnancy, with a higher MTCT rate of 5.9% [37]. In Zimbabwe, however, the majority (61.04%) knew their HIV status before pregnancy, and the MTCT rate was much lower at 1.55% [47]. Similarly, in Brazil, most pregnant women (84%) were diagnosed with HIV before or during pregnancy, and the MTCT rate was 2% [26]. In Dessie Town, 59.7% of women received ARV before pregnancy, and the MTCT rate was 3.8% [40]. A study conducted in Latin America revealed that delayed maternal HIV diagnosis not only increased the risk of MTCT but also worsened the clinical and immunological outcomes of HIV‐infected children [48]. Failure to diagnose maternal HIV infection before delivery often results in missed opportunities for PMTCT prophylaxis and early infant diagnosis [49]. Early HIV diagnosis in women, ideally before pregnancy, is a cornerstone for effective PMTCT and is key to achieving the goal of eliminating vertical transmission.

Women who did not live within the catchment area of PMTCT services were more likely to have seropositive infants compared to those residing within the catchment area [AOR = 4.74 (1.17, 20.63)]. Long travel distances to health facilities have been shown to increase the risk of loss to follow‐up and home delivery [50, 51], while also reducing the likelihood of timely initiation of PMTCT regimens [52]. When mothers and their HEI fail to receive adequate ART treatment, ANC follow‐up, and facility‐based delivery, the risk of vertical HIV transmission increases substantially [40].

Women who knew their HIV status before pregnancy were less likely to have seropositive infants compared to those whose status was determined in the postpartum period [AOR = 0.08(0.009,0.84)]. This finding is consistent with studies conducted in East and West Gojjam Zones and Southern Ethiopia [37, 41]. Women knew their status before pregnancy had a higher chance of early initiation of ART (before pregnancy or during first trimester pregnancy), which leads to HIV viral load suppression [53]. A longer duration of ART during pregnancy has been shown to be associated with suppressed viral load at delivery and a lower rate of MTCT of HIV [54]. Indeed, women who initiated ART prior to conception and maintained viral suppression achieved virtually zero risk of MTCT [55].

Women who did not take ART were more likely to have HIV‐positive infants compared to those who received ART [AOR = 6.82 (1.33, 38.73)]. This finding is consistent with studies conducted in Dessie town, Ethiopia, Kenya, Cameron, and Vietnam [28, 33, 34, 36, 40]. Initiation of ART before or during pregnancy is associated with reduced maternal viral load [53]. When the maternal viral load is suppressed, the risk of MTCT is significantly decreased [56].

Women who gave birth at health facilty were less likely to have sero‐positive infants as compared to those who delivered at home [AOR = 0.08 (0.01, 0.66)]. This finding is consistent with studies conducted in Dire Dawa City, the Amhara region, Southern Ethiopia, Uganda, and East Africa [25, 32, 36, 38, 40, 41]. Infants delivered at health facilities were more likely to receive Nevirapine prophylaxis, which significantly reduces the risk of MTCT [40]. In contrast, infants born at home often missed ART prophylaxis at birth, and those who did not receive ART prophylaxis were more likely to become HIV‐positive [32].

Children who received Nevirapine prophylaxis were less likely to be HIV‐positive compared to those who did not receive Nevirapine prophylaxis [AOR = 0.14 (0.03, 0.65)]. This finding is consistent with studies conducted in various parts of Ethiopia, other African countries, and Vietnam [25, 28, 32, 34, 36, 38, 40].

## 5 Limitation

This study has some limitations that should be considered when interpreting the findings. First, as a retrospective study, several important independent variables were not routinely recorded in medical records. Consequently, factors that might influence MTCT of HIV but were not documented were excluded from the analysis. Second, some of the study variables had wide confidence intervals, which may be attributed to the relatively small sample size. A larger sample could have provided more precise estimates.

## 6 Conclusion

The rate of vertical HIV transmission in this study was relatively high. Delivery at a health facility, infant Nevirapine prophylaxis, and maternal HIV diagnosis before pregnancy were found to be protective factors against MTCT. In contrast, residing outside the PMTCT catchment area and failure to initiate maternal ART were identified as significant risk factors.

To reduce vertical transmission, it is essential to expand PMTCT services, strengthen maternal HIV diagnosis during the preconception period, and ensure consistent provision of maternal ART and infant Nevirapine prophylaxis. Promoting facility‐based delivery should also remain a priority.

## Author Contributions

**Amare Genetu Ejigu:** conceptualization, data curation, formal analysis, funding acquisition, investigation, methodology, project administration, resources, software, supervision, validation, visualization, writing – original draft, writing – review and editing. **Tilahun Degu:** formal analysis, funding acquisition, investigation, software, validation, writing – original draft, writing – review and editing. **Ahmed Fentaw Ahmed:** conceptualization, data curation, software, validation, visualization, writing – review and editing. **Abathun Temesgen:** data curation, formal analysis, investigation, methodology, software, supervision, validation, writing – original draft, writing – review and editing. **Almaw Genet Yeshiwas:** conceptualization, formal analysis, methodology, software, validation, writing – original draft, writing – review and editing. **Abebaw Molla Kebede:** conceptualization, formal analysis, methodology, supervision, visualization, writing – original draft, writing – review and editing. **Gashaw Melkie Bayeh:** data curation, funding acquisition, investigation, methodology, software, visualization, writing – original draft, writing – review and editing. **Chalachew Yenew:** data curation, formal analysis, project administration, software, supervision, validation, writing – original draft, writing – review and editing. **Asaye Alamneh Gebeyehu:** data curation, funding acquisition, methodology, supervision, validation, writing – review and editing. **Biresaw Wassihun Alemu:** conceptualization, data curation, formal analysis, methodology, software, supervision, writing – original draft, writing – review and editing. **Zeamanuel Anteneh Yigzaw:** formal analysis, investigation, methodology, software, supervision, writing – review and editing. **Habitamu Mekonen:** formal analysis, methodology, resources, software, supervision, visualization, writing – review and editing. **Tewodros Worku Bogale:** conceptualization, formal analysis, investigation, methodology, resources, software, validation, visualization, writing – review and editing. **Rahel Mulatie Anteneh:** data curation, formal analysis, methodology, project administration, validation, visualization, writing – review and editing. **Anley Shiferaw Enawgaw:** data curation, formal analysis, methodology, software, validation, writing – review and editing. **Getasew Yirdaw:** funding acquisition, investigation, methodology, software, supervision, validation, writing – review and editing. **Roza Belayneh Desalegn:** conceptualization, funding acquisition, methodology, project administration, resources, supervision, validation, visualization, writing – review and editing. **Berhanu Abebaw Mekonnen:** formal analysis, investigation, methodology, resources, supervision, validation, visualization, writing – review and editing. **Meron Asmamaw Alemayehu:** conceptualization, data curation, funding acquisition, investigation, methodology, resources, supervision, validation, writing – original draft, writing – review and editing. **Sintayehu Simie Tsega:** data curation, formal analysis, investigation, methodology, resources, supervision, validation, visualization, writing – review and editing.

## Ethics Statement

This study was conducted from February to April 2023. Ethical clearance was obtained from the Institutional Review Committee of Injibara University (Reference No: 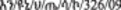). The objectives and purpose of the study were communicated to the administrators of the respective health institutions, and permission for data collection was secured before the study commenced. The research adhered to the principles of the Declaration of Helsinki. Data were extracted from mother–infant registration records; no personal identifiers were collected, confidentiality was strictly maintained, and the information was used solely for the purpose of this study. As the data were secondary and fully anonymized, individual informed consent was not required, in accordance with ethical guidelines and the approval granted by the Institutional Review Committee.

## Consent

The authors have nothing to report.

## Conflicts of Interest

The authors declare no conflicts of interest.

## Transparency Statement

The lead author Amare Genetu Ejigu affirms that this manuscript is an honest, accurate, and transparent account of the study being reported; that no important aspects of the study have been omitted; and that any discrepancies from the study as planned (and, if relevant, registered) have been explained.

## Data Availability

After obtaining ethical clearance from the zonal and district health offices and public health institutions, an agreement was made not to publish the raw data retrieved from the women and HEI registers. However, the datasets generated and analyzed during the current study are available from the corresponding author upon reasonable request.
